# Influence of established and subjectively perceived as well as evaluated individual characteristics on the utilization of mental health services among individuals with depressive disorders: protocol of a longitudinal study examining how to supplement the “behavioral model of health services use” and on need-congruent use of mental health services

**DOI:** 10.1186/s12888-021-03065-w

**Published:** 2021-02-02

**Authors:** Anna Katharina Reinhold, Julia Louise Magaard, Anna Levke Brütt

**Affiliations:** 1 Department of Health Services Research, Faculty of Medicine and Health Sciences, Carl von Ossietzky University Oldenburg, Oldenburg, Germany; 2 Department of Medical Psychology, Center for Psychosocial Medicine, University Medical Centre Hamburg-Eppendorf, Hamburg, Germany

**Keywords:** Major depressive disorder, Behavioral model of health services use, Subjective need, Utilization behavior, Mental health care, Longitudinal data

## Abstract

**Background:**

Approximately one out of every three people in Germany who meets the diagnostic criteria for major depression has contact with mental health services. Therefore, according to treatment guidelines, two thirds of all individuals with depression are insufficiently treated. In the past, the subjective perspective of people who (do not) make use of mental health services has been neglected. Factors related to the use of health services are described in Andersen’s Behavioral Model of Health Services Use (ABM). The aim of this study is to supplement operationalizations of subjectively perceived and evaluated individual characteristics in the ABM and to evaluate whether the supplemented model can better explain mental health services use in individuals with depression than established operationalizations.

**Methods:**

A representative telephone study with two measurement points will be conducted. In an explanatory mixed-methods design, qualitative interviews will be added to further interpret the quantitative data. A nationwide sample scoring 5 or more on the Patient Health Questionnaire (PHQ-9) will be recruited and interviewed via telephone at T0 and 12 months later (T1). Data on established and subjective characteristics as well as mental health service use will be collected. At T1, conducting a diagnostic interview (Composite International Diagnostic Interview, DIA-X-12/M-CIDI) enables the recording of 12-month diagnoses according to DSM-IV-TR criteria. Ideally, *n* = 768 datasets will be available and analyzed descriptively by means of regression analysis. Up to *n* = 32 persons who use or do not use depression-specific health services incongruent with their objective or subjective needs will be interviewed (face-to-face) to better explain their behavior. In addition, theories of non-need-based mental health service use are developed within the framework of the grounded theory-based analysis of the qualitative interviews.

**Discussion:**

The study intends to contribute to the theoretical foundation of health services research and to specify the characteristics described in the ABM. Thus, after completion of the study, a further sophisticated and empirically tested model will be available to explain mental health services. The identified modifiable influencing factors are relevant for the development of strategies to increase mental health service use in line with the objective and subjective needs of individuals with depression.

**Supplementary Information:**

The online version contains supplementary material available at 10.1186/s12888-021-03065-w.

## Background

In Germany, the 12-month prevalence of diagnosed unipolar depression is 10.6% in women and 4.8% in men [1]. In addition to the load of symptoms, depression is associated with impaired quality of life and functioning [2, 3]. Due to its frequency and complex consequences depression is a challenge for the health care system. In Germany, outpatient, day-care and inpatient services are being provided for individuals suffering from depression [4], and an established guideline specifies which treatment is appropriate [5]. Especially for people with moderate or severe depressive symptoms and people with persistent mild depressive symptoms (> 2 weeks), treatment is indicated. In these cases, psychotherapy and/or pharmacotherapy [5] would be appropriate, according to the guidelines. However, analyses from the German Health Interview and Examination Survey for Adults (DEGS) show that approximately one out of every three (34.6%) who met the diagnostic criteria for major depression in the previous 12 months used mental health services [6]. There is a relevant gap between need and mental health services use that has to be bridged.

An established generic model, the “Behavioral Model of Health Services Use” by Andersen (ABM) [7], can be used to further investigate utilization behavior [8]. The model, which has been continuously developed since the 1960s, describes various determinants of health behavior and health-related outcomes. The most recent version [7] focuses on predisposing factors, enabling factors and need as essential contextual and individual predictors of health behavior and thus health services use.

Contextual characteristics describe environmental variables such as the presence and accessibility of facilities. Age and gender, educational status, occupational status, ethnicity, social relations and health beliefs are established predisposing individual characteristics [8]. Likewise, at the individual level, income, insurance status, presence of a permanent general practitioner (GP), means of transport, travel time and waiting time are established enabling factors, while symptoms and subjective needs are established need factors [8]. In this model, the subjective perception of those affected is considered in personal health beliefs and perceived needs. In empirical studies, however, these subjective characteristics have only rarely been investigated [9].

A systematic review [10] focusing on help-seeking individuals with major depression indicates that men (e.g., [11]), young adults and elderly people (e.g., [12, 13]), ethnic minorities (e.g., [14]) and people with lower levels of education (e.g., [13]) have a higher risk of not receiving professional health care (predisposing). With regard to need factors, positive correlations were reported between mental health service use and the severity of symptoms (e.g., [11]), duration of disease (e.g., [14]) and presence of comorbid anxiety disorder (e.g., [15]). Health beliefs have only been investigated in 5 of 39 included studies [10]. Similarly, Roberts et al. [16] found only two studies considering stigma and no study considering mental health literacy as an enabling factor for service use for individuals with mental disorders. However, a recent study showed that biomedical causal beliefs are related to need and intention to seek help in individuals with untreated mental illness [17]. In addition, a qualitative synthesis conducted to understand help-seeking behavior in depression emphasizes the importance of subjective beliefs [18]. In addition to the lack of research with regard to subjective beliefs, the cross-sectional design of most of the studies limits their value for explaining mental health service use [10, 16].

In summary, Germany provides a range of mental health services, but only one in three individuals experiencing symptoms of major depression seeks professional help. Therefore, it is of particular interest to better explain the process of mental health service use and especially take into account subjective beliefs and perceived need.

## Methods

### Objectives

**Fig. 1:**
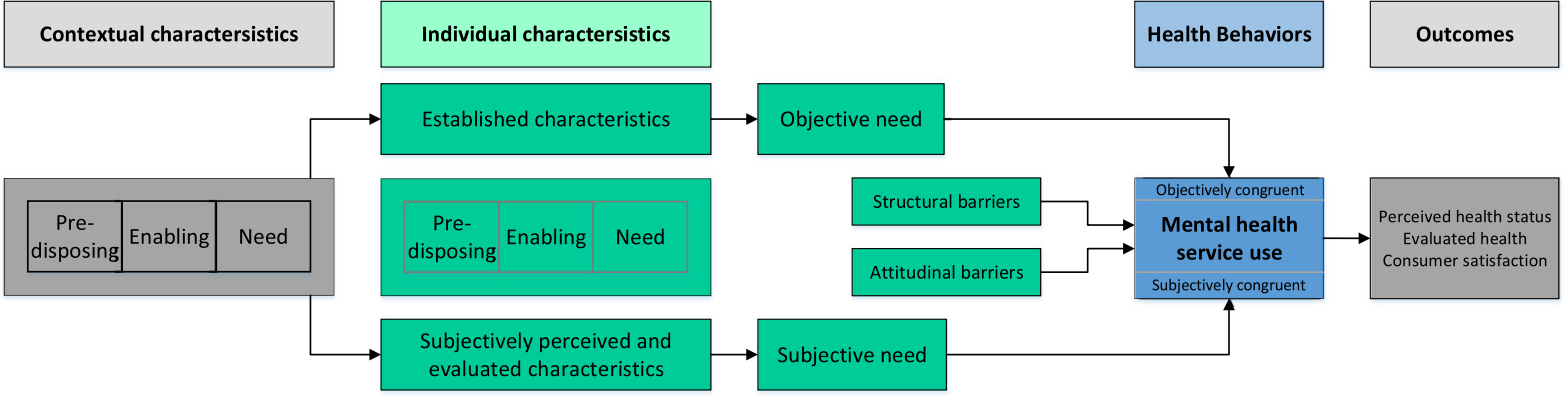
Addition of the “Behavioral Model of Health Services Use” (Anderson, 2008) on the basis of own preliminary work

A longitudinal study will be conducted to determine whether subjectively perceived and evaluated individual characteristics supplemented in the ABM (see Fig. 1) influence the mental health services use of individuals with depression. Furthermore, how objective needs, resulting from the diagnosis and the corresponding guidelines, and subjective needs, resulting from subjectively perceived and evaluated characteristics, can foster mental health service use will be examined.

The following research questions will be answered:

1. Does supplementing subjectively perceived and evaluated individual characteristics (subjective need, illness beliefs) in the ABM contribute to a better explanation of mental health service use in people with depressive symptoms?

2. What is the proportion of individuals (not) using mental health services incongruent with their objective or subjective need?

3. How do individuals deal with incongruency regarding subjective needs, objective needs and mental health service use?

### Study design

A prospective, longitudinal study is planned: A representative telephone sample will be drawn, and interviews will take place at T0 (inclusion in the study) and T1 (12 months after inclusion in the study). At T1, additional qualitative interviews in a selected subsample will be conducted. An explanatory sequential design will be used for combining quantitative data with data from qualitative interviews [19].

### Recruitment of participants

The participants will be recruited and interviewed via telephone by the independent social research institute USUMA GmbH. The representative sample will be generated from landline and mobile numbers according to the Association of German Market and Social Research Agencies (ADM) dual-frame approach [20]. The proportion of mobile numbers in the total sample will be approximately 40%. The selection framework includes all telephone numbers that can be used within Germany [20], which are processed using an adaptive generation method [21]. Generated transferable telephone numbers are contacted up to 10 times. The first call will be made on weekdays between 4:30 and 9:00 pm or on Saturdays, as the availability of the contact or target person is best during these periods. If the call is not answered, follow-up calls will be made on different days of the week at different times of the day to increase the probability of reaching the contact or target person. While the mobile phone sample is a priori regarded as a sample of persons, the landline sample is initially a household sample, which is then converted into a sample of persons implementing the Kish-Selection-Grid technique [22].

The interviewers will inform potential participants by telephone about the purpose and procedure of the study, about the collection and processing of personal data (contact data, research data) and about their right to withdraw. Subsequently, they will obtain and document oral consent to participate. After giving informed consent, interviewees will answer a short screening questionnaire (Patient Health Questionnaire (PHQ-9), [23]). Interviewees who are aged 18 or older and who reach a score of at least 5 on the PHQ-9, corresponding to mild depressive symptomatology, will be included in the study. The interview will continue or a date for the interview will be scheduled. Recruitment is completed as soon as the University of Oldenburg research team (UORT) has received the T0 dataset from USUMA GmbH.

To reach participants at T1, the name, address/e-mail address and telephone number of the respondents (contact data) will be recorded and linked to the research data via a pseudonymization list. The list will be administered by USUMA GmbH in accordance with data protection regulations.

Prior to the start of the second wave of the survey (T1), USUMA GmbH will transfer contact data (name as well as address or e-mail address) without a pseudonymization key to the UORT. The UORT will contact participants by mail/email prior to T1 to send material for the diagnostic interview (answer lists [24, 25]) and detailed information about the planned qualitative face-to-face interviews. Contact data will be deleted by the UORT as soon as all participants will have been contacted.

At T1, USUMA GmbH will contact T0 participants again via telephone. At the end of the telephone interview, USUMA GmbH will ask participants for consent for an additional qualitative face-to-face interview conducted by UORT. Contact and research data will be transferred to UORT for those who consented. Written informed consent will then be given in person before the qualitative interview begins. Theoretical sampling will be used as a criterion for selecting participants for qualitative face-to-face interviews. Based on the quantitative data, participants having used mental health services in the past 12 months and a) objective need or b) subjective need only and participants not having used mental health services in the past 12 months and c) objective or d) subjective need only will be selected. Thus, this selection will take into account different facets of incongruency between objective and subjective needs [26]. In the first step, 16 cases will be selected on the basis of the quantitative data, 4 for each possible incongruence between subjective and objective needs. The interviews will be analyzed, and then an additional eight cases will be selected. The research team will then decide on the necessity of further interviews. A maximum of 32 interviews will be conducted.

### Measurements

Measurements were piloted in a cross-sectional mixed-method design with depressive patients (*n* = 205) from different settings (general practitioner care, outpatient specialist care, inpatient specialist care) and members of self-help groups (unpublished observations; involved researchers: ALB, JLM). Established individual as well as subjectively perceived and evaluated individual characteristics were operationalized and collected. In addition, participants specified their mental health services used within the last 12 months due to “mental problems (e.g., depressive symptoms)”. Based on the results, measurements for this longitudinal study were chosen. Table 1 depicts the schedule of enrollment and assessment of the study. Further information, which contains references to the development and/or psychometric properties of the instruments for the German and/or English version is available in the Additional file 1. Furthermore, all adaptations are indicated (see Additional Table 1).

**Table 1:** Schedule of enrollment and assessment

	STUDY PERIOD		
Timepoint	T0	T1	T1
	Baseline Telephone interview	12 months Telephone interview	14 months Qualitative interview
Informed consent	X		X
**Established characteristics**			
Predisposing			
Age	X		
Sex	X		
Family status	X	Changes are queried	
Migration background	X		
Enabling			
Socioeconomic status	X	Changes are queried	
Health insurance	X	Changes are queried	
Presence of a general practitioner	X	Changes are queried	
Need			
Subjective health (SF-8)	X	X	
Depressivity (PHQ-9)	X ^a^	X	
Risk of mental comorbidity	X	X	
**Complementary characteristics**			
Predisposing			
Subjective illness perception (IPQ-Brief)	X	X	
Enabling			
Barriers (checklist according to the World Mental Health Survey, SELFI, SSOSH)	X^b^	X	
Need			
Perceived need for care (GUPI)	X	X	
Mental health services use	X	X	
Diagnostic interview (DIA-X-12/M-CIDI)		X	
Conditions and reasons for (non-) utilization			X

^a^ serves as eligibility screen at T0, ^b^ only SELFI and SSOSH will be surveyed at T0

#### Quantitative data collection

Telephone interviews will be conducted by USUMA GmbH. Answers of participants will be directly recorded in an online database. The UORT receives pseudonymized datasets after completion of T0 and in the process of T1, including a final dataset. Depression scores will already be collected during screening with the PHQ-9 [23, 27] at T0. At T1, in addition to the PHQ-9, a diagnostic interview will be conducted with the Composite International Diagnostic Interview (DIA-X-12/M-CIDI [24, 25];). The computer-aided interview enables the recording of 12-month diagnoses according to DSM-IV-TR on the basis of algorithms [24, 25]). Only sections E (depressive disorders and dysthymic disorders) and F (mania and bipolar affective disorders) will be surveyed.

Recording of established individual characteristics: Data on sociodemographic characteristics (age, family status, sex, partnership situation, education, job situation, ethnicity, size of household, income/financial situation, federal state) as well as type of health insurance and the existence of a GP will be collected. Based on selected characteristics, the socioeconomic status index (SES-Index, [28]) can be calculated.

Subjective health will be measured using the SF-8 short version [29–31] of the SF-36 questionnaire [32, 33]. With a total of 8 items, this version reflects the two sum scales of physical health and mental health. The PHQ-9 [23, 27] for recording depressive symptoms has already been described above.

The risk of mental comorbidity will be assessed with the help of four screening instruments. Comorbidities that will be considered are generalized anxiety disorder (Generalized Anxiety Disorder Scale-7 (GAD-7), [34, 35]), alcohol use disorder (Alcohol Use Disorders Identification Test - Consumption (AUDIT-C), [36–38]) and criteria A (Patient Health Questionnaire 15 (PHQ-15), [23, 39]) and B (Somatic Symptom Disorder Scale-12 (SSD-12), [40, 41]) of somatic symptom disorder. The risk of mental comorbidity is represented by a cumulative score. Persons in whom no mental comorbidity is present will be assigned a score of 0, and persons in whom all three comorbidities are positively screened will be assigned a score of 3.

Recording of supplemented subjectively perceived and evaluated individual characteristics: Depression beliefs will be collected with a 9-item short version [42] of the Illness Perception Questionnaire-R (IPQ-R, [43]). The short version is based on selected items from the psychometrically tested German translation [44]. Since there will be no reliable diagnosis of the respondents at the time of the survey, the term “illness” will be replaced by the term “psychological complaints”.

The perceived barriers will be collected through lists that include attitude-related barriers (e.g., “I wanted to deal with the problem on my own.”) and structural barriers (e.g., “The waiting time was too long.”). A distinction is made between reasons for not having sought help (list 1), reasons for not having found help (list 2) and reasons for having terminated treatment prematurely (list 3). The lists are based on the approach of the World Health Organization (WHO) World Mental Health Survey [45] and were adapted according to the results of the pilot study. The reasons given by more than 25% of the respondents in the pilot study will be recorded.

Self-stigmatization as a possible barrier to mental health service use is recorded by means of the Self-Stigma Scale of Seeking Help (SSOSH, [46]). The scale consists of 10 items and is designed for application to people who have not yet sought professional help (e.g., “It would make me feel inferior to ask a therapist for help.”). Participants with previous therapy experience will be instructed to imagine a forthcoming appointment with a psychologist when answering the questions.

Self-identification of the affected persons as mentally ill is recognized as an important precondition in the process of help-seeking [47] and will be assessed by means of the self-identification of having a mental illness (SELFI) scale [48–50].

An adapted translation of the General-practice Users Perceived-need Inventory (GUPI, [51]), a self-assessment tool based on the Perceived Need for Care Questionnaire (PNCQ, [52]), will be used to record perceived care needs. The GUPI measures the perceived need for information on mental disorders, medication, counseling or psychotherapy, social interventions and skills training (e.g., employability, self-care). A version adapted on the basis of the results of the pilot study will make it possible to determine the subjective need independent of receiving mental health care. Each category can be assessed with “I would like to use this offer of help.”, “I do not want to use this offer of help.”, “I already use this offer of help and I have also wanted to use it.” and “I am already using this help offer, but I did not want to use it.”.

The recording of mental health service use is based on the procedure in the DEGS supplementary mental health examination (DEGS-MHS, [53]). The questions will be specified for four areas of care (outpatient care, inpatient care, GP care, low-threshold care). At T0, lifetime, 12-month and current mental health service use will be recorded, and at T1, 12-month use only will be recorded. Furthermore, satisfaction with the current or past treatment will be surveyed. In addition, the date of the first and last contact with the respective area of care is also recorded at T1. The treatment (type, frequency, duration, satisfaction) will also be described at T1. Although the retrospective recording of health services use will be exposed to memory effects, the method that is used and already has been tested in the pilot study (inquiring about salient utilization events, e.g., hospital stays, beginning of psychotherapy or psychopharmaceutical use over a period of 12 months) will improve the accuracy of the measurement [54].

#### Qualitative data collection

Qualitative face-to-face interviews will be conducted with a subsample by the UORT. The interviews will be based on a guide that will be developed by the UORT. The main topics will be subjective concepts of disease and treatment, subjectively perceived barriers and facilitators for using mental health services and stigma experiences. The interviews will be recorded electronically and will be transcribed verbatim.

### Analysis

#### Quantitative analysis

For the statistical calculations, the mental health services use reported retrospectively at T1 will be converted into a dichotomous variable. Additional analyses will be carried out with dichotomous variables related to specific mental health services (outpatient care, inpatient care, GP care, low-threshold care). Based on the guidelines for unipolar depression [5], an objective need for psychopharmacotherapy or psychotherapy will be operationalized by mild and moderate depression in the past 12 months, while an objective need for combination therapy will be operationalized by severe depression in DIA-X-12/M-CIDI [24, 25]. Subjective need for care will be operationalized via the categories “unsatisfied need” and “satisfied need” as a result of the GUPI [51].

The quantitative evaluation will be preceded by a nonresponder analysis. A hierarchical logistic regression analysis will be performed to answer the main research question. The dependent variable will be mental health service use (dichotomous measuring level: yes/no). Independent variables will be the collected individual characteristics. First, the set of established individual characteristics will be included in the regression (10 predictors), followed by the set of complementary subjectively perceived and evaluated individual characteristics (7 predictors) (see Table 2). A likelihood quotient test will be used to check whether the complete model with all predictors better predicts mental health service use than the reduced model with established individual characteristics.

**Table 2:** Overview of operationalization and measurement instruments

	predictors (***n*** = 17)	
	Established individual characteristics (***n*** = 10)	Subjectively perceived and evaluated individual characteristics (***n*** = 7)
Predisposing	(1) age (2) family status (3) sex (4) migration background	depression beliefs: IPQ-Brief [42, 44] (1) identity (2) time course (3) consequences (4) personal control (5) treatment control (6) coherence
Enabling	(5) socioeconomic status: SES-Index, [28] (6) health insurance (7) GP	barriers (descriptive): checklist according to the World Mental Health Survey [45], SELFI [48–50], SSOSH [46]
Need	(8) Subjective health: SF-8 [29–31] (9) depressivity: DIA-X-12/M-CIDI [24, 25] (10) risk of mental comorbidity index [23, 34–41]	(7) perceived need for care: GUPI [51]
	**Outcome (*n = 1)***	
Health service use	mental health service use according to DEGS [53]	

Descriptive statistics will be used to account for the proportion of people who use mental health services congruent or incongruent to their needs (research question 2). The diagnosis of a depressive disorder will be interpreted as objective need, and data gathered in the GUPI [51] will be interpreted as subjective need. Mental health service use will be considered retrospectively for the past 12 months (T1). Thus, the proportions of objective and subjective congruent as well as incongruent mental health service use can be described.

### Qualitative analysis

To answer research question 3, individual case analyses of contrasting cases and then comparative analyses based on the grounded theory approach will be conducted [26]. Within the framework of grounded theory, theoretical coding will be applied. For this analysis approach, transcripts will be read and coded in teams of two. Codes will be combined into categories. Using axial coding, the categories and their relationships to other categories will be examined in a second step. Selective coding as the third step of the analysis will focus on the key categories and prepare the final theory formation. In this process, relationships and interactions between the themes will be examined. The coding and analysis of the interviews will be carried out with support from MAXQDA software [55].

### Sample size

**Fig. 2:**
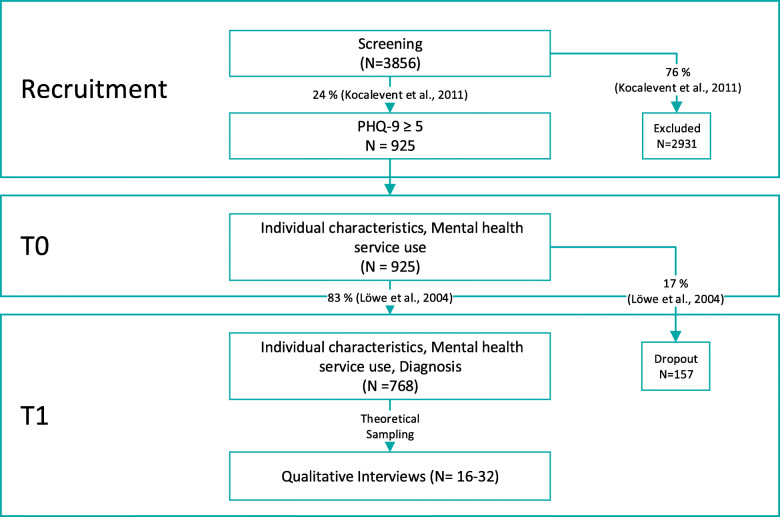
Data collection process. Recruitment will continue until *n* = 925 datasets are available at T0. The number of cases at T1 is an approximate target number that will be realized depending on the dropout

To answer research question 1, regression models will be evaluated. To avoid overfitting, we used the Harrells [56] heuristic for the limiting number of cases. Based on 17 variables to be included in the regression models, we aim for at least *n* = 170 persons at T1 who have used mental health services in the previous 12 months. Further sample size planning considered drop-out [57, 58], data on PHQ-9 specificity regarding the detection of depression [59] and DEGS results [1, 6]. Recruitment will be terminated by USUMA GmbH as soon as datasets of *n* = 925 subjects are available at T0. If this number of cases will be reached at T0, it is expected that approximately *n* = 768 persons (83%) can be interviewed at T1 [58] (see Fig. 2). Of those, 652 individuals could fulfil diagnostic criteria for depression [59], and 215 (33%) could report mental health services use [1, 6]. With an assumed error of up to 20%, between 172 and 258 persons receiving mental health care should be in the sample at T1.

## Discussion

The study outlined here is intended to provide insight into the theoretical basis of mental health services use and to contribute to specifying the characteristics described in the ABM. To the best of our knowledge, this is the first representative, longitudinal study that collects diagnoses by conducting diagnostic interviews and investigates the association between subjectively perceived and evaluated characteristics and the mental health service use of people with depressive disorders. Thus, after completion of the study, a more sophisticated and empirically tested model will be available to explain the mental health service use of people with depression. Our findings, although derived from the German context, will be valuable internationally, as evidence regarding the role of subjective depression beliefs and perceived need in the process of help-seeking and mental health services use is weak [10, 16]. Our supplemented version of the ABM can be examined in other contexts; moreover the ABM explicitly includes contextual characteristics so that further research can take these into account.

### Strengths and weaknesses

The research questions posed in our study are supported by the results of extensive preliminary work. To obtain meaningful results, we will only use validated instruments, some of which we have evaluated in the pilot study. By using an explanatory mixed-method design, selected cases will be analyzed in depth. Thus, incongruencies in the mental health service use of individuals with depression can not only be described but also explained.

A limitation of our study regards recruitment. It can be assumed that patients with severe depression symptoms do not answer the phone at all or interrupt the conversation in between, causing a selection bias. However, due to the severe symptoms, it is also difficult if not impossible to interview these patients in a personal interview, either in a private or clinical setting. It is also conceivable that the screening instrument (PHQ-9) may react more sensitively due to coronavirus-related limitations. Therefore, people who would not have been included before the crisis could be included in our study.

An analysis of mental health service use is essential for improving access to mental health care. As noted above, two-thirds of people with major depression remain untreated [1]. The results of the study outlined here add to the theoretical foundation of mental health services research. They can contribute to the identification of barriers in the process of mental health service use, the development of theoretically based strategies to improve the mental health service use of people with depression congruent with objective needs as well as subjective needs, and the improvement and patient-centeredness of mental health services.

## Supplementary Information

**Additional file 1: Table S1.** Schedule of enrollment and assessment providing references and adaptations; An extension of Table 1, which contains references to the development and/or psychometric properties of the instruments for the German and/or English version. Furthermore, all adaptations are indicated.

## Data Availability

The datasets generated and analyzed during the current study will not be publicly available to protect patient confidentiality. Selected variables will be available from the corresponding author upon reasonable request.

## Abbreviations

ABM: Andersen’s Behavioral Model of Health Services Use
ADM: Association of German Market and Social Research Agencies
AUDIT-C: Alcohol Use Disorders Identification Test – Consumption
DEGS: German Health Interview and Examination Survey for Adults
DEGS-MHS: German Health Interview and Examination Survey for Adults Supplementary Mental Health examination
DFG: German Research Foundation
DIA-X-12/M-CIDI: Diagnostisches Expertensystem für psychische Störungen/Munich-Composite International Diagnostic Interview
DSM-IV-TR: Diagnostic and Statistical Manual of Mental Disorders
GAD-7: Generalized Anxiety Disorder Scale-7
GDPR: General Data Protection Regulation
GP: General Practitioner
GUPI: General-practice Users Perceived-need Inventory
IPQ-Brief: Illness Perception Questionnaire Brief
IPQ-R: Illness Perception Questionnaire-R
PHQ-9: Patient Health Questionnaire-9
PHQ-15: Patient Health Questionnaire-15
PNCQ: Perceived Need for Care Questionnaire
SELFI: Self-Identification as Having Mental Illness-Scale
SES-Index: socioeconomic status Index
SF-8: Shortform-8-Questionnaire
SF-36: Short Form Health Survey
SSD-12: Somatic Symptom Disorder Scale-12
SSOSH: Self-Stigma Scale of Seeking Help
UORT: University of Oldenburg research team
