# Supplementary material for: Influence of established and subjectively perceived as well as evaluated individual characteristics on the utilization of mental health services among individuals with depressive disorders: protocol of a longitudinal study examining how to supplement the “behavioral model of health services use” and on need-congruent use of mental health services
Source: BMC Psychiatry. 2021 Feb 2;21:68. doi: 10.1186/s12888-021-03065-w (PMC7851814; doi:10.1186/s12888-021-03065-w)
Supplement: Supplementary file 1 — Additional file 1: Table S1. Schedule of enrollment and assessment providing references and adaptations; An extension of Table 1, which contains references to the development and/or psychometric properties of the instruments for the German and/or English version. Furthermore, all adaptations are indicated. [file 12888_2021_3065_MOESM1_ESM.docx]

***Additional table 1 Schedule of enrollment and assessment providing references and adaptations***

|  | | | | Study period |  |  |
| --- | --- | --- | --- | --- | --- | --- |
| Timepoint | | | | T0 | T1 | T1 |
|  | English version^a^ | German version^a^ | Adaptations | Baseline  Telephone interview | 12 months  Telephone interview | 14 months  Qualitative  interview |
| Variables/Measures |  |  |  |  |  |  |
| Informed consent |  |  |  | X |  | X |
| **Established characteristics** |  |  |  |  |  |  |
| Age |  |  |  | X |  |  |
| Sex |  |  |  | X |  |  |
| Family status |  |  |  | X | Changes are queried |  |
| Migration background |  |  |  | X |  |  |
| Socioeconomic status |  |  |  | X | Changes are queried |  |
| Health insurance |  |  |  | X | Changes are queried |  |
| Presence of general  practitioner |  |  |  | X | Changes are queried |  |
| Subjective health (SF-8) | Ware et al. 2001 | Ellert et al. 2005, Schulz et al. 2009 | none | X | X |  |
| Depressivity (PHQ-9) | Kroenke et al. 2001 | Gräfe et al. 2004 | none | X ^b^ | X |  |
| Risk of mental comorbidity  index (GAD-7, AUDIT-C,   PHQ-15, SSD-12) | *GAD-7:* Spitzer et al. 2008; *AUDIT-C:* Saunders et al. 1993, Bush et al. 1998;  *PHQ-15:* Kroenke et al. 2002; *SSD-12:* n.a. | *GAD-7:* Löwe et al. 2008; *AUDIT-C:* Moehring et al. 2019;  *PHQ-15:* Gräfe et al. 2004;  *SSD-12:* Toussaint et al. 2016, Toussaint et al. 2017 | none | X | X |  |
| **Complementary characteristics** |  |  |  |  |  |  |
| Subjective illness   perception (IPQ-Brief) | Moss-Morris et al. 2002, Broadbent et al. 2006 | Glattacker et al. 2009 | Since there will be no reliable diagnosis of the respondents at the time of the survey, the term "illness" will be replaced by the term "psychological complaints". | X | X |  |
| Barriers (checklist   according to the World  Mental Health Survey,   SELFI, SSOSH) | *Checklists:* Andrade et al. 2014; *SELFI:* Evans-Lacko et al. 2019;  *SSOSH:* Vogel et al. 2006 | *Checklists:* Andrade et al. 2014; *SELFI:* Schomerus et al. 2012; Schomerus et al. 2019;  *SSOSH:* n.a. | *Checklists* were adapted according to the results of the pilot study. The reasons given by more than 25 percent of the respondents in the pilot study will be recorded.  *Barriers that will not be collected:*  “You didn’t need help anymore. “, “The therapist or counselor left or moved away.”, “The policies were a hassle.”, “You moved.”, “Your health insurance would not pay for more treatment. “, “You got better.”, “You were not getting better.”, “You had bad experiences with the treatment providers.”, “You were concerned about what people would think if they found out you were in treatment.”, “You were treated badly or unfairly.”, “You felt out of place.”, “Your family wanted you to stop.”  *Additional/adapted barriers:*  “I could not afford the money for it.”, “I have not found a practitioner/therapist.”, “The waiting time was too long.”, “The practitioner/therapist did not see any need/possibility to treat me.”, “The practitioner/therapist has not spent enough time.”, “The problem disappeared by itself and I no longer needed any help.”, “I was treated differently by my environment than before the mental health service use.”, “I was concerned that my family/friends/employers might find out that I was in treatment.” | X^c^ | X |  |
| Perceived need for care   (GUPI) | McNab et al. 2005 | n.a. | Response options were adapted according to the results of the pilot study making it possible to determine the subjective need independent from receiving mental health care. Each category can be assessed with "I would like to use this offer of help.", "I do not want to use this offer of help.", "I already use this offer of help and I have also wanted to use it." and "I am already using this help offer, but I did not want to use it.". | X | X |  |
| Mental health service use | n.a. | Jacobi et al. 2013 | Response options were adapted on the basis of the pretest results.  *Inpatient facilities:*  “Psychiatric, psychotherapeutic or psychosomatic clinic or ward”, “Psychosomatic rehabilitation”, “Psychiatric, psychotherapeutic or psychosomatic day clinic”, “Other inpatient facilities namely”  *Outpatient facilities:*  “Psychiatric, psychotherapeutic or psychosomatic outpatient clinic”, “(Socio-)psychiatric service”, “Psychiatrist/Neurologist”, “Psychotherapist”, “General practitioner (for mental problems)”, “Counseling centers for” (to be specified), “Other outpatient facilities namely”  *Low-threshold services:*  “Self-help organizations”, “Telephone counseling”, “Internet-based services, e.g., forums, apps, online programs (e.g., of health insurance companies)”, “Other low-threshold services namely” | X | X |  |
| Diagnostic interview (DIA-X-12/M-CIDI) sections E and F | Wittchen 1994 | Wittchen & Pfister 1997 | none |  | X |  |
| Conditions and reasons for (non-) utilization |  |  |  |  |  | X |

^a^ only provided for validated measurements; ^b^ serves as eligibility screen at T0; ^c^ only SELFI and SSOSH will be surveyed at T0.
